# Impact of Anaerobic Fermentation Liquid on Bok Choy and Mechanism of Combined Vitamin C from Bok Choy and Allicin in Treatment of DSS Colitis

**DOI:** 10.3390/foods14050785

**Published:** 2025-02-25

**Authors:** Junhui Pan, Kaitao Peng, Roger Ruan, Yuhuan Liu, Xian Cui

**Affiliations:** 1 State Key Laboratory of Food Science and Resources, Engineering Research Center for Biomass Conversion, Ministry of Education, Nanchang University, Nanchang 330047, China; panjunhui@ncu.edu.cn (J.P.); pkt1811826@163.com (K.P.); 2 Center for Biorefining and Department of Bioproducts and Biosystems Engineering, University of Minnesota, St. Paul, MN 55108, USA; ruanx001@umn.edu; 3 Chongqing Research Institute of Nanchang University, Chongqing 402660, China

**Keywords:** anaerobic fermentation liquid, Bok choy, vitamin C, inflammatory enteritis, intestinal flora

## Abstract

In the context of pollution-free waste treatment, anaerobic fermentation liquid (AFL), a prominent by-product of biogas engineering, has emerged as a focal point in contemporary research. Concurrently, vitamin C, an active compound abundant in fruits and vegetables, possesses extensive application potential. The development of efficient extraction processes and the utilization of its biological activities have garnered significant attention from researchers. This study investigated the impact of AFL on the growth and vitamin C content of Bok choy through field trials of varying concentrations of AFL. The results indicated that the growth characteristics of Bok choy exhibited a concentration-dependent trend with increasing AFL dosage, with the highest yield observed in the AFL-2 group (8.43 kg/m^2^). Additionally, with the increase in the concentration of the AFL application, the vitamin C content in Bok choy exhibited a trend of initially increasing and then decreasing, reaching its highest value (70.83 mg/100 g) in the AFL-1 group. Furthermore, response surface methodology was employed to optimize the microwave-assisted organic solvent extraction process of vitamin C, revealing that the optimal conditions for microwave-assisted extraction using a 2% citric acid solution were as follows: a microwave power of 313 W, a microwave time of 1.3 min, and a liquid-to-solid ratio of 16.4:1 *v*/*w*, achieving a vitamin C extraction rate of 90.77%. Subsequent mechanistic studies on colitis repair demonstrated that the combination of vitamin C and allicin significantly enhanced the ability of intestinal microorganisms to ferment and degrade complex carbohydrates in colitis-afflicted mice, thereby alleviating intestinal inflammation, markedly reducing bacterial invasion signals on intestinal epithelial cells, and decreasing the risk of intestinal infection. This study provides a valuable perspective for the harmless utilization of agricultural waste, and provides a theoretical basis and technical support for the high-value utilization of natural active ingredients.

## 1. Introduction

Anaerobic fermentation liquid (AFL), a by-product of biogas engineering, exhibits high yields and is rich in essential trace elements and nutrients such as iron, zinc, manganese, amino acids, and vitamin B [1,2,3]. It possesses the characteristics of an efficient organic fertilizer. Compared to chemical fertilizers, AFL offers a higher concentration of readily available nutrients and a diverse nutrient profile, making it more easily absorbed by plants [4]. Furthermore, the application of AFL positively regulates the soil environment, promoting water and nutrient retention, thereby enhancing crop yields [5,6]. Li et al. demonstrated that a 3% concentration of AFL was the most beneficial for cucumber growth and disease resistance without causing soil salinization [7]. Bok choy (*Brassica rapa* subsp. *chinensis*), a widely cultivated vegetable, is rich in essential nutrients such as vitamin C, vitamin K, and minerals critical for human health [8,9]. However, limited research has explored the effects of AFL on Bok choy yield and its vitamin C content. Moreover, traditional solvent extraction methods for vitamin C extraction have drawbacks, including a low product purity and a susceptibility to oxidation [10]. Consequently, researchers have focused on developing new extraction methods to improve yields and purity. Microwave-assisted extraction (MAE) has emerged as a preferred technique due to its high extraction efficiency, reduced heating time, and energy savings [11].

Inflammatory bowel disease (IBD) is a chronic inflammatory condition affecting the gastrointestinal tract, characterized by mucosal structural damage, intestinal microbial imbalance, and systemic biochemical abnormalities [12]. Studies indicate that vitamin C (ascorbic acid) and allicin, commonly found in fruits and vegetables, can reduce the levels of inflammatory markers [13]. Vitamin C plays a crucial role in cell metabolism and brain function, acting as an antioxidant to counteract reactive oxygen species and active nitrogen produced during normal cell metabolism [14,15]. It also modulates the inflammatory response [16]. Therefore, vitamin C holds potential as a therapeutic agent for IBD. Allicin, a sulfite ester widely used in food as an active compound, has been shown to regulate gut microbiota, reducing inflammatory responses and barrier damage [17,18,19]. Shi et al. demonstrated that allicin can improve DSS-induced ulcerative colitis by inhibiting the MAPKs-PPAR-γ/NF-κB/AP-1/STAT-1 signaling pathway [20]. However, the combined effects and mechanism of vitamin C and allicin on colitis remain understudied, and whether they exhibit synergistic effects is unclear.

This study aims to investigate the impact of AFL as a substitute for chemical fertilizers on the growth quality, yield, and vitamin C content of Bok choy. Additionally, vitamin C will be extracted from Bok choy using microwave-assisted extraction. Moreover, a mouse model of colitis induced by dextran sulfate sodium (DSS) will be established to explore the mechanisms and immune system effects of vitamin C and allicin combination therapy in treating colitis. This research seeks to elucidate the molecular and physiological pathways through which these compounds positively influence inflammatory enteritis. The ultimate objective is to promote the application of AFL as a fertilizer substitute, to clarify its effects on the growth of Chinese cabbage and the physiological activity of vitamin C, and to provide new insights for the harmless treatment of agricultural waste and the intervention of the high value utilization of active ingredients.

## 2. Materials and Methods

### 2.1. Materials

The variety of Bok choy used in this experiment was “Siji” Bok choy. The fertilizer used in this experiment was urea (about 46.6% nitrogen). The anaerobic digestion protoplasm used in the experiment was sourced from Ganzhou Ruiyuan Biotechnology Co., LTD (Ganzhou, China). Dextran sulfate sodium (DSS) was purchased from Sigma Chemical Co., LTD (St. Louis, MO, USA). The remaining chemical reagents were analytically pure and purchased from Xilong Science Co., LTD (Chengdu, China).

### 2.2. Experimental Design of AFL Application in Alternative Fertilizers

The experimental field was located at Ganzhou Ruiyuan Biotechnology Co., in Ganzhou City, Jiangxi Province, China (24°58′ N, 115°3′ E). AFL after 60 days of age-effect treatment was used in this study. The physical and chemical properties of the treated AFL and the basic physicochemical properties of the tested soil have been reported in our previous study [21]. Among them, the specific physicochemical parameters of treated AFL, including total nitrogen, total salt, total phosphorus, electric conductivity, odor concentration, and the content of potassium, magnesium, calcium, copper, zinc, arsenic, and selenium have been listed in Table S1. Furthermore, the total organic compound and humic acid content of AFL were quantified by following the methodologies detailed in the referenced literature [22,23]. The resultant data are presented in Table 1.

#### 2.2.1. Experimental Design

(1) Experimental Design for Planting: Bok choy seeds were sown using a direct seeding method. Each planting plot measured 1.6 m in width and 2.2 m in length, covering a total area of 3.52 m^2^, with a plant spacing of 15 cm to ensure adequate space for photosynthesis and growth. A 0.5 m wide isolation strip was established between the plots. These isolation strips consisted of soil beds, each containing three ridges separated by ditches into which AFL was directly irrigated. The experiment comprised five treatment groups, each replicated three times, resulting in a total of 15 plots. To ensure the fairness and repeatability of the experiment, the arrangement of plots followed a randomized principle determined by on-site randomization.

(2) Treatment Group Design: Based on previous experiments, one group served as a blank control group (BC). Only organic fertilizer (primarily biogas residue) was applied as a base fertilizer, with no additional fertilizers applied during the growth period. In the chemical fertilizer group (CF), only urea (approximately 46.6% N) was used. Three AFL groups (AFL-0.5, AFL-1, AFL-2) received only AFL treatments, with the total nitrogen content being 0.5, 1, and 2 times that of the CF group, respectively. The specific dosages for each treatment are detailed in Table 2.

(3) Fertilization Method: Base fertilizer was applied prior to planting, consisting of organic fertilizer mixed thoroughly into the soil before seed sowing. During the entire growth cycle of the Bok choy, topdressing was conducted three times—at the seedling promotion stage, the seedling strengthening stage, and the harvest stage. The topdressing material was the treated AFL, and foliar fertilizers were sprayed according to the designated amounts for each experimental group. The environmental conditions were strictly controlled and maintained consistently across all treatments and controls.

#### 2.2.2. Determination of Bok Choy

After the first fertilization and harvest, the Bok choy was sampled randomly three times. In each plot, three sample squares of 1 m^2^ were selected to observe the growth and take samples for laboratory processing and analysis. We measured the plant height (vertical distance from the soil surface to its top), number of leaves (≥1 cm), the fresh weight of the whole Bok choy, and the fresh weight of the edible parts (root removed) of each Bok choy in the selected plot. After the digestion of the Bok choy samples, the vitamin C content was measured by HPLC (1260, Agilent, Santa Clara, CA, USA) with an Agilent HC-C18(2) column, a flow rate of 0.7 mL/min, a detection wavelength of 245 nm, a column temperature of 25 °C, and an injection volume of 20 µL [24].

### 2.3. Optimal Extraction Experiment of Vitamin C

A microwave synthesator (DC8789, CEM Co., LTD. Matthews, NC, USA) was used to assist in the extraction of vitamin C from Bok choy harvested after the application of AFL. The extraction conditions were optimized by Box–Behnken experiments. Microwave power, microwave time, and the liquid–solid ratio were selected as the three main factors affecting the extraction rate of vitamin C. The three-factor and three-level test was designed, and the optimal extraction conditions of vitamin C were determined with the extraction rate of vitamin C as the response value. The specific reaction conditions are shown in Table 3. A similar method was used to extract allicin from biogas slurry garlic obtained in the laboratory [21].

### 2.4. Animal Experiment

#### 2.4.1. Experimental Group Design and Sample Collection

A total of 60 C57BL/6J male mice of 6 weeks old were purchased from Shanghai Yaokang Biotechnology Co., Ltd. (Shanghai, China). and raised in a temperature-controlled room (22 ± 2 °C). After 3–7 days of adaptive feeding, the mice were randomly divided into 10 groups according to their body weight, namely: the negative control group (G1), the DSS model group (G2), and the positive control groups: DSS: cyclosporine A group (G3), DSS: vitamin C group (G4), DSS: allicin low-dose group (G5), DSS: allicin medium-dose group (G6), DSS: allicin high-dose group (G7), DSS: allicin low-dose + vitamin C group (G8), DSS: allicin medium-dose + vitamin C group (G9), and DSS: allicin high-dose + vitamin C group (G10). The Group G1 mice drank water normally every day during the test period; Group G2–G10 mice were treated with 3% DSS on day 1–9, and drank water normally on day 9–12. G3–G10 mice were orally administered 40 mpk cyclosporine A, 300 mpk vitamin C, 10 mpk allicin, 20 mpk allicin, 40 mpk allicin [25,26], 10 mpk allicin +300 mpk vitamin C, 20 mpk allicin +300 mpk vitamin C, and 40 mpk allicin +300 mpk vitamin C daily from day 1 to day 12. Among them, the administration volume of the G2–G7 group mice was 10 μL/g × mouse weight (g); and the G8–G10 group mice were administered with each drug alone, the drug concentration was unchanged, and the drug volume was doubled. The order of administration was consistent for each dose and grouped. The health status of the mice was observed every day during the test period, the body weight of mice was recorded, and the feces of the mice were collected for determining subsequent DAI scores. On the 12th day of the experiment, all mice were anesthetized before their necks were broken, and biological samples were collected. The colon contents of the mice were collected and frozen in liquid nitrogen, and then stored at −80 °C for subsequent 16s intestinal flora sequencing; the colonic anterior tissues were isolated and fixed in 4% PFA for subsequent colon morphological observation (light microscope, UV-2600, Shimadzu Co., Ltd., Kyoto, Japan) [27]; and the colonic tissues of 2–3 cm were collected and frozen in liquid nitrogen, and then stored at −80 °C for subsequent cytokine detection and analysis.

#### 2.4.2. Disease Activity Index (DAI) Analysis

The DAI referred to the research method used in previous research [28], and was slightly modified to comprehensively evaluate weight loss, stool characteristics, and stool occult blood. The specific scoring methods were as follows: weight loss score: 0 (weight gain or unchanged), 1 (weight loss within 5%), 2 (weight loss between 5 and 10%), 3 (weight loss between 10 and 15%), 4 (weight loss above 15%); stool character score: 0 (normal stool), 1–2 (loose stool), 3 (watery stool); and stool occult blood: 0–1 (normal), 2–3 (occult blood), 4 (hemorrhage/massive hemorrhage).

#### 2.4.3. RNA Extraction and RT qPCR

We weighed 100 mg colon tissue and extracted total RNA with an RNA extraction kit (R711-01, Nanjing Vazyme Biotechnology Co., Ltd. Nanjing, China). The specific operational method was carried out according to the instructions for use provided by the manufacturer. The extracted total RNA was reverse transcribed into cDNA based on the instructions of the reverse transcription kit (R433-01, Nanjing Vazyme Biotechnology Co., Ltd.). The cDNA obtained after inversion was subjected to fluorescence quantitative PCR (real-time fluorescence quantitative PCR instrument, ABI Quant Studio 5 Q5, Thermo Fisher Scientific, Waltham, MA, USA) according to the instructions of the dye-based fluorescence quantitative reagent kit (Q712-02, Nanjing Vazyme Biotechnology Co., Ltd. Nanjing, China). The results of relative gene expression were expressed by 2^−ΔΔCT^ according to Livak et al. [29]. The genes tested in the assay included interleukin-1β (IL-1β), interleukin 6 (IL-6), tumor necrosis factor α(TNF-α), and the internal reference gene GAPDH (Table 4).

#### 2.4.4. Fecal DNA Extraction and 16S DNA Sequencing

We weighed 0.3 g of a thawed mouse feces sample and placed it into a centrifuge tube containing extraction lysate, and grinded it at a 60 Hz frequency. Nucleic acids were extracted from stool samples using the OMEGA Soil DNA Kit (Omega Bio-Tek, Norcross, GA, USA). The DNA from the extracted samples was quantified using Nanodrop (Thermo Scientific, NC2000).

A 50 ng DNA template (enzyme linked immunosorbent assay analyzer, Rayto RT-6100, Rayto USA) was used to amplify the V3–V4 region of 16S rDNA in combination with the upstream primer 524F (5′-barcode+TGYCAGCCGCCGCGGTAA-3′) and downstream primer 958R (5′-YCCGGTTGAVTCCAATT-3′). The target fragments of the PCR amplification products were isolated via 1.5% agarose gel electrophoresis and subsequently recovered using the AxyPrep DNA Gel Extraction Kit (Shanghai Personal Biotechnology Co., Ltd. Shanghai, China). The quantification of the PCR products was performed with the Quant-iT PicoGreen dsDNA Assay Kit (Invitrogen, Carlsbad, CA, USA). Following this, samples were pooled according to the required sequencing depth for each sample, and libraries were prepared and sequenced on the Illumina NovaSeq platform [30].

The microbiomes of the mouse feces samples were analyzed for biological information using QIIME2. The original sequence data were decoded by the demux plug-in, primer excision was performed by the cutadapt plug-in, and finally, quality filtering, denoising, splicing, and chimera removal were performed on the sequence data. Taxonomic information corresponding to each ASV was obtained by comparing ASV feature sequences with reference sequences in the Greengenes database. ASVs with abundance values of less than 0.001% of the total number of samples sequenced were removed, and abundance matrices with rare ASVs removed were used for subsequent series of analyses.

Firstly, QIIME2 software (2022.11) was used to draw sparse curves to judge whether the sequencing depth of each sample met the standard, and then the α diversity of the microorganisms in the sample were calculated, including Chao1 index, Shannon index, and Simpson index. Beta diversity was measured by UniFrac distance and visualized by principal coordinate analysis (PCoA) using R software (v3.2.0) and QIIME2 software (2022.11). According to the ASV classification and taxonomic status identification results, the number of microbial groups contained in the different samples at each taxonomic level was compared, and then the above data were drawn into histograms by R software to visually compare the number of taxonomic units in different samples at the same level. KEGG function enrichment of the microbial functions in the different samples was carried out by PICRUSt2 (v2.5.2, Phylogenetic Investigation of Communities by Reconstruction of Unobserved States) software.

### 2.5. Statistical Analysis

The experimental method was repeated three times, and the data were organized using Microsoft Excel 2016. IBM SPSS 22.0 was utilized for difference and correlation analysis, while Origin 2021 was used for graphing. For group comparisons, univariate analysis of variance followed by the Tukey–Kramer test were applied, and in this study, lowercase letters, superscripted or not, indicate significant differences between groups under the same indicator at *p* < 0.05.

## 3. Results

### 3.1. Effects of Different Fertilization Treatments on Bok Choy

#### 3.1.1. Effects of Different Fertilization Treatments on Plant Height and Leaf Number of Bok Choy

**Figure 1:**
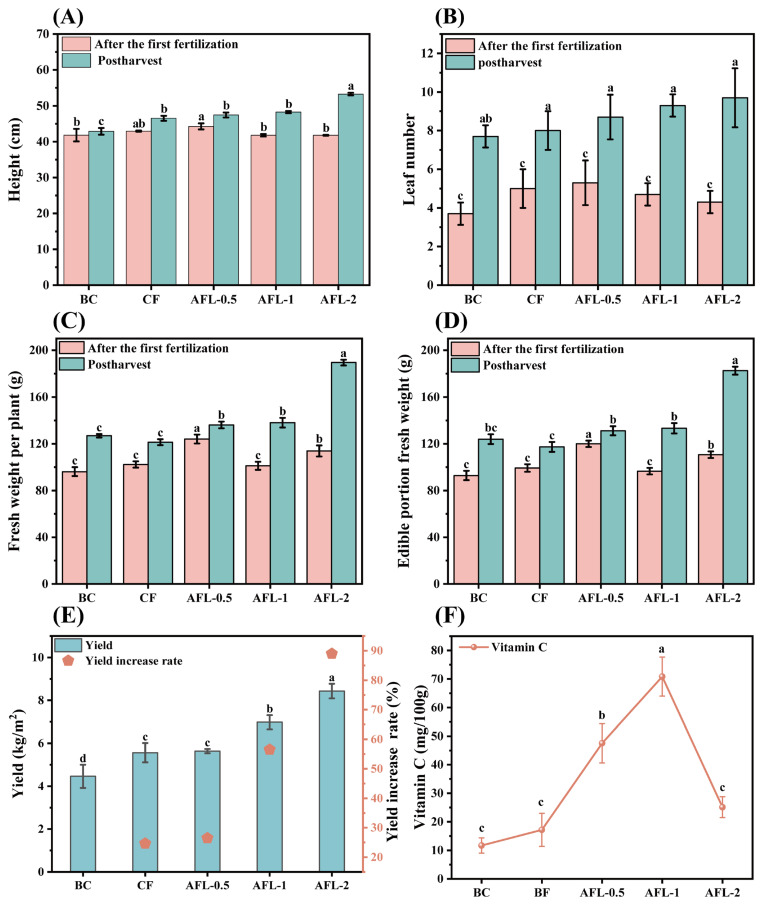
Effects of different fertilization treatments on Bok choy. (**A**) Height, (**B**) leaf number, (**C**) fresh weight per plant, (**D**) edible portion fresh weight, (**E**) yield, and (**F**) vitamin C content. Different lowercase letters indicate the different significance between treatments (*p* < 0.05).

The growth and development of Bok choy are closely associated with its final yield, with fertilization strategy playing a pivotal role. Figure 1A,B illustrate the dynamic changes in plant height and leaf number under various fertilization treatments. Following the first fertilization, the plant height sequence was AFL-0.5 > CF > BC > AFL-1 = AFL-2, with the AFL-0.5 group reaching 44.25 cm, which was significantly higher than the BC, AFL-1, and AFL-2 groups (*p* < 0.05). The AFL-0.5 group also exhibited the highest average leaf number, 5.3, surpassing the CF group. Notably, leaf number declined with increasing levels of AFL application. This enhanced growth performance in the AFL-0.5 group is attributed to the slow-release nature of AFL, which provides a continuous and steady nutrient supply during the seedling stage, thereby supporting more consistent plant development [31]. In contrast, the rapid nutrient release from chemical fertilizers, which resulted in elevated soil nutrient concentrations, inhibited growth in the AFL-1 and AFL-2 groups. This suggests that while AFL offers a gradual nutrient release beneficial for plant growth, excessive or improper application of anaerobic fertilizers may have detrimental effects on Bok choy development [32].

After harvest, the Bok choy plant height increased with a higher AFL usage, surpassing the CF group. The AFL-2 group had the tallest plants at 53.25 cm, followed by AFL-1 at 48.24 cm. The AFL-1 group significantly differed from the others, showing increases of 24.2%, 14.5%, 12.2%, and 12.7% compared to BC, CF, AFL-2, and AFL-0.5 (*p* < 0.05). The AFL-2 group exhibited the most significant height increase from fertilization to harvest, followed by AFL-1. Leaf number trends mirrored plant height, with AFL-2 and AFL-1 leading, as follows: AFL-2 > AFL-1 > AFL-0.5 > CF > BC. The average leaf number increased significantly after the first fertilization, reaching 9.7 in AFL-2 and 9.3 in AFL-1 (*p* < 0.05). Overall, Bok choy effectively utilized AFL, which promoted growth more than chemical fertilizer, aligning with the findings of Liang et al. [4].

#### 3.1.2. Effects of Different Fertilization Treatments on the Yield of Bok Choy

The impact of different fertilization treatments on the yield of Bok choy is illustrated in Figure 1C–E. As depicted in Figure 1C,D, following the initial fertilization, varying concentrations of AFL had differential effects on the fresh weight of Bok choy. Notably, the AFL-0.5 group exhibited the highest fresh weight per plant (124.1 g), which was significantly higher than that observed in the BC and CF groups (*p* < 0.05). Additionally, AFL-1 demonstrated a particularly pronounced effect on root growth compared to AFL-0.5 and AFL-2, likely due to its balanced nutrient composition providing optimal conditions for root development.

Post-harvest analysis revealed the following order of fresh weight per plant: AFL-2 > AFL-1 > AFL-0.5 > BC > CF. Specifically, plants treated with AFL-2 achieved a fresh weight of 189.47 g, representing a significant increase of 49.3% and 56.1% compared to the BC and CF groups, respectively (Figure 1C). The superior nutrient content of AFL, particularly in terms of nitrogen, phosphorus, potassium, amino acids, vitamin B, and plant growth regulators, may account for this enhanced growth promotion [3]. Moreover, AFL is rich in organic matter, which can improve soil structure and nutrient availability, offering a more comprehensive nutrient profile than conventional fertilizers [4]. This is especially advantageous for nutrient-demanding crops like Bok choy, which require adequate nitrogen for optimal leaf development and photosynthetic efficiency [32]. Furthermore, higher concentrations of AFL positively influence root development, enhancing the crop’s ability to withstand adverse environmental conditions.

Figure 1E illustrates the final yield of Bok choy under different fertilization treatments. Consistent with previous observations, the AFL-2 group achieved the highest final yield at 8.43 kg/m^2^. The yield increased in a concentration-dependent manner with AFL, attributed to the provision of a wide range of essential nutrients in bioavailable forms, thereby positively impacting growth and yield. These findings suggest that AFL can serve as an effective and sustainable alternative fertilizer for nutrient-demanding crops such as Bok choy.

#### 3.1.3. Effect of Different Fertilization Treatments on Vitamin C Content in Bok Choy

The effects of different fertilization treatments on the vitamin C content of Bok choy are shown in Figure 1F. As can be seen from the figure, the concentration of vitamin C in the AFL-1 group increased significantly by 504.9% compared with BC, 312.2% compared with CF, 182.2% compared with AFL-2, and 49.1% compared with AFL-0.5 (*p* < 0.05). While both AFL-1 and AFL-0.5 can lead to a significant increase in vitamin C relative to BC, AFL-2 showed a similar response to CF, suggesting that AFL-2 may not provide an additional benefit over CF under test conditions. The enhanced vitamin C synthesis observed under AFL treatment may be attributed to the nutrient-rich component of AFL, which appears to promote the better absorption of essential elements such as iron, a key cofactor in vitamin C biosynthesis [33]. However, high concentrations of AFL, particularly AFL-2, should be approached with caution, as they may lead to the accumulation of nitrates in Bok choy and impair vitamin C synthesis [34,35,36], consequently degrading the overall quality of the crop.

Based on the aforementioned analysis, the judicious application of AFL as a substitute for chemical fertilizers (AFL-1) can significantly enhance the growth and development of Bok choy, while concurrently increasing both its final yield and vitamin C content. Consequently, Bok choy from the AFL-1 group was selected as the source material for subsequent vitamin C extraction.

### 3.2. Optimization of Extraction Process of Vitamin C from Bok Choy

The experimental design and results of Box–Behnken are shown in Table S2. The results of the vitamin C extraction rate of the 17 groups of experiments were fitted, and a ternary quadratic function with vitamin C extraction rate as the dependent variable, and the microwave power, microwave time, and microwave solid–liquid ratio as the independent variables was obtained:

$$Y=88.47-4.12A-2.12B+2.84C+6.93\mathrm{AB}-0.135\mathrm{AC}-0.9075\mathrm{BC}-5.54A^{2}-6.86B^{2}-4.38C^{2}$$

where Y is the extraction rate of vitamin C, A is the microwave power, B is the microwave time, and C is the liquid–material ratio.

The results of the Quadratic vs. 2FI model corresponding to the vitamin C extraction rate are shown in Table S3. It can be seen from the table that the *p* value in the model is <0.0001, indicating that the model can be used to calculate the vitamin C extraction rate significantly. The *p* value of the missing item was 0.7297 > 0.05, indicating a high degree of fitting between the model and the experiment, and the accidental factors in the experiment did not significantly affect the extraction rate of vitamin C. The variance R^2^ in the model was 0.9954, indicating that 99.54% of the change in the extraction rate of vitamin C was caused by the response variable. The correction coefficient of the model was Adjusted R^2^, and the predicted variance was 0.9760, which was close to Adjusted R^2^ (0.9895). This all indicated that the fitted model was of high accuracy and the model could interpret and predict the results of the response to the vitamin C extraction rate. In addition, the Adeq Precision of the model was 37.6709 > 4, which indicated that the model had a high resolution. The *p* values of each factor on the extraction rate of vitamin C were all less than 0.05, which indicated that the microwave power, microwave time, and solid–liquid ratio had significant effects on the extraction rate of vitamin C. The influence of the microwave power and liquid–solid ratio on the extraction rate of vitamin C were greater than that of microwave time.

**Figure 2:**
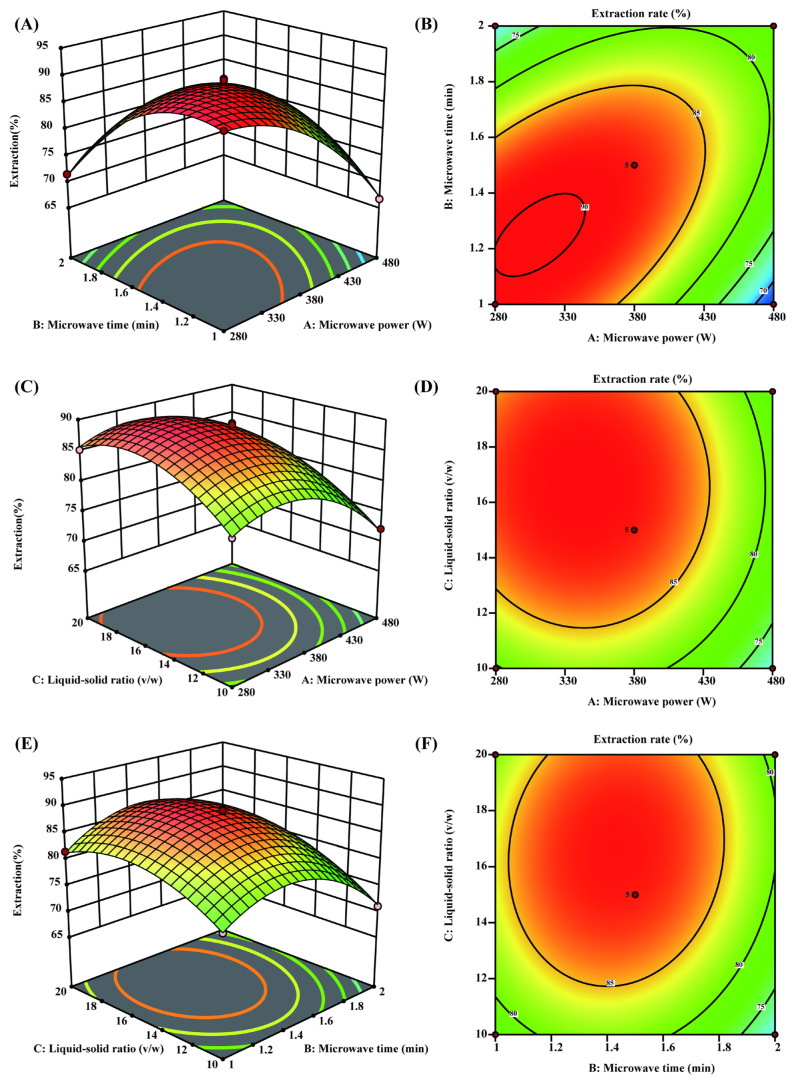
Effects of microwave power, microwave time, liquid–solid ratio, and their interaction with the extraction rate of vitamin C. (**A**) The three-dimensional response surface diagram and (**B**) the contour diagram of microwave power and microwave time and their interactions with the extraction rate of vitamin C; (**C**) the three-dimensional response surface diagram and (**D**) the contour diagram of microwave power and liquid–solid ratio and their interactions with the extraction rate of vitamin C; (**E**) the three-dimensional response surface diagram and (**F**) the contour diagram of microwave time and liquid–solid ratio and their interactions with the extraction rate of vitamin C.

In the interaction, the *p* value of AB < 0.01, the *p* value of BC < 0.05, and the *p* value of AC > 0.05, indicating that microwave power and microwave time interaction (AB) and microwave time and liquid–solid ratio interaction (BC) had high and significant effects on the extraction rate of vitamin C. The effect of microwave power on the liquid–solid ratio interaction (AC) was not significant. Similar conclusions can also be drawn from the three-dimensional response surface plot and contour plot of the interaction of the three factors on the extraction rate of vitamin C (Figure 2).

Based on the model, the optimal microwave treatment conditions for maximizing the extraction rate of vitamin C are as follows: a microwave power of 313 W, a microwave duration of 1.3 min, and a liquid–solid ratio of 16.4:1. Under these conditions, the extraction rate of vitamin C can reach 90.77%. To validate these optimal conditions, three parallel verification experiments were conducted, with results summarized in Table 5. The average extraction rate of vitamin C was 90.68%, and the purity reached 89.57%. These experimental outcomes fall within the acceptable margin of error, confirming the reliability of the response surface model. Moreover, compared to the commonly used ultrasonic-assisted extraction method for vitamin C, microwave-assisted extraction significantly reduced the reaction time and enhanced the extraction efficiency [37,38].

### 3.3. Effects of Vitamin C/Allicin Combined Treatment on Colitis

#### 3.3.1. Impact of Vitamin C/Allicin Combined Treatment on the Phenotype of Colitis

**Figure 3:**
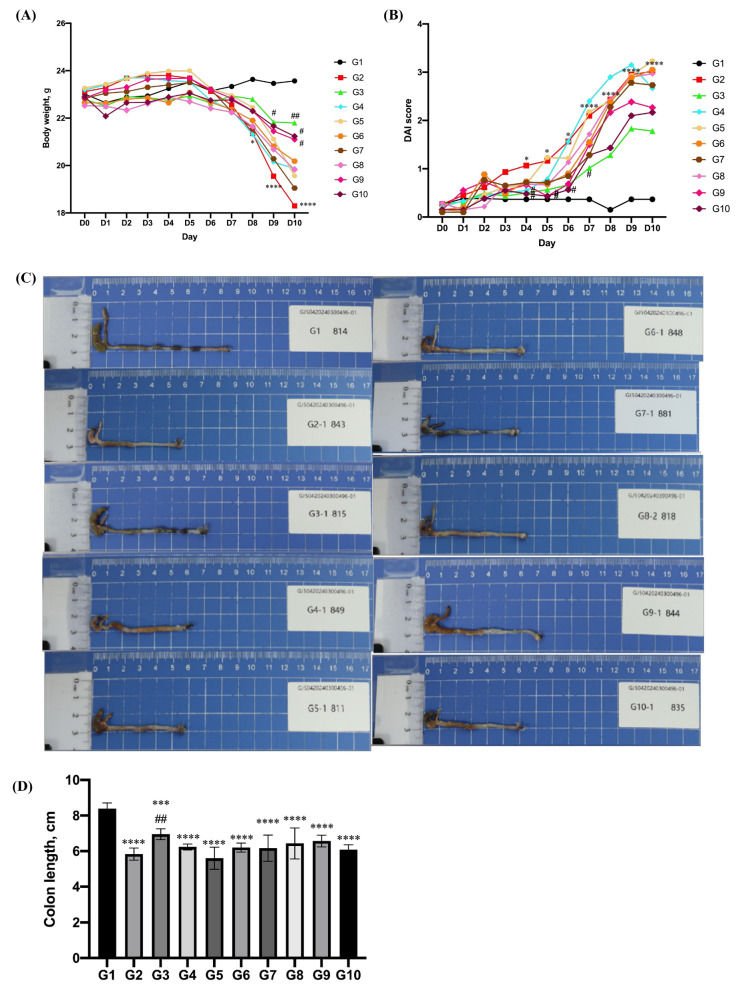
Effects of vitamin C/allicin combined treatment on body weight (**A**), DAI score (**B**), and colon length (**C**, **D**) of mice induced by DSS. Note: “*” represents a significant difference compared with group G1, where * indicates *p* < 0.05, *** indicates *p* < 0.001, and **** indicates *p* < 0.0001; “#” represents a significant difference compared with group G2, where # indicates *p* < 0.05, and ## indicates *p* < 0.01.

In order to explore whether vitamin C/allicin can alleviate the inflammatory response in the colon, mice were treated with 3% DSS in their drinking water for 9 days, and vitamin C/allicin was supplemented orally. It has been demonstrated in previous studies that colitis in mice can lead to symptoms such as body weight loss, diarrhea, rectal bleeding, and the shortening of the colon [39,40]. Consequently, the body weight and disease activity index (DAI) scores were monitored on a daily basis, and the length of the colon in mice was measured at the end of the experiment to observe the symptoms of colitis in mice. The results are presented in Figure 3. During the first 8 days of the experiment, there was no significant difference in body weight among the different groups. Treatment with cyclosporin A could significantly suppress the body weight loss induced by DSS in mice on days 9–10 (*p* < 0.05). The combined treatment with vitamin C/allicin could prevent the body weight loss caused by DSS in a dose-dependent manner (*p* < 0.05), yet the effects of allicin or vitamin C administered alone were not significant. Moreover, during the first 4 days of the experiment, no significant differences were observed in the DAI scores among the various groups. Treatment with the immunosuppressant cyclosporin A could significantly inhibit the elevation of the DAI scores induced by DSS on days 5 and 8 (*p* < 0.05). The combined treatment with vitamin C/allicin could significantly suppress the increase in the DAI scores caused by DSS on days 5–7 (*p* < 0.05), while the inhibitory effects of allicin or vitamin C alone were not remarkable.

In DSS-induced colitis, colon shortening has been verified as an indicator of the severity of inflammation [41]. Compared to the control group, the length of the colon in the DSS-treated mice was significantly decreased, indicating that treating mice with 3% DSS in their drinking water for 9 days could successfully induce colitis; that is, the model establishment was successful. Treatment with cyclosporin A could significantly inhibit the shortening of the colon induced by DSS (*p* < 0.05). Treatment with vitamin C, allicin, or the combined treatment with vitamin C/allicin could, to a certain extent, suppress the shortening of the colon induced by DSS.

#### 3.3.2. Regulation of Vitamin C/Allicin Combined Treatment on the Promotion of Pro-Inflammatory Cytokines

**Figure 4:**
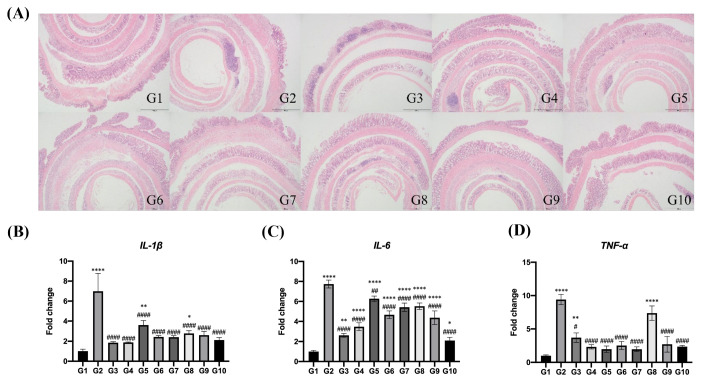
Effects of vitamin C/allicin combined treatment on tissue morphology (A) and the mRNA expression of IL-1β (B), IL-6 (C), and TNF-α (D) in the colon of mice induced by DSS. Note: Scale bars: 100 μm. “*” represents a significant difference compared with group G1, where * indicates *p* < 0.05, ** indicates *p* < 0.01, and **** indicates *p* < 0.0001; “#” represents a significant difference compared with group G2, where # indicates *p* < 0.05, ## indicates *p* < 0.01, and #### indicates *p* < 0.0001.

The congestion, necrosis, and edema of colonic tissues constitute the typical manifestations of colitis. A multitude of studies have demonstrated that in the mouse model of colitis induced by dextran sulfate sodium (DSS), severe pathological symptoms such as mucosal ulceration and the destruction of crypt structures emerge. As illustrated in Figure 4A, in Group G1 (the control group), the surface of the colonic mucosal layer of mice was smooth, and the epithelial tissues of the intestinal glands were orderly and tightly arranged. In contrast to the control group, the colonic mucosal layer of DSS-treated mice was severely damaged, with the crypt structures disrupted and a pronounced infiltration of inflammatory cells. When compared with Group G1, the colonic injuries in mice of the vitamin C/allicin intervention groups were relatively attenuated, and the crypt structures were more intact. Notably, in Group G8, a slight infiltration of inflammatory cells was observed, while in Group G9, no infiltration of the inflammatory cells was detected, signifying the most efficacious treatment outcome. This indicates that high-concentration vitamin C/allicin exerts a favorable protective effect on the colons of mice, and its intervention can substantially mitigate the colonic tissue damage in mice afflicted with colitis and ameliorate the inflammatory grade of colitis.

The aberrant secretion of pro-inflammatory cytokines represents the principal etiological factor underlying chronic inflammatory pathologies, including acute lung injury and inflammatory bowel disease (IBD), and may potentially serve as an ancillary carcinogenic factor [42,43]. Pro-inflammatory cytokines, namely TNF-α, IL-1β, and IL-6, play pivotal roles in the inflammatory process, precipitating tissue damage and exacerbating colitis [44]. Elevated levels of these cytokines have been reported in patients with ulcerative colitis (UC), Crohn’s disease (CD), and colorectal cancer (CRC) [45]. Research has elucidated that the alterations in DSS-induced colitis manifest most conspicuously in the middle region of the large intestine and the distal segment terminating at the rectum [46]. Hence, to further dissect the protective effect of the combined treatment of vitamin C/allicin on DSS-induced colitis in mice, we quantified the relative gene expression levels of inflammation-related cytokines IL-1β, IL-6, and TNF-α in the middle and posterior segments of the colon. As depicted in Figure 4B–D, in comparison with the control group, DSS treatment led to a significant augmentation in the relative gene expression levels of IL-1β, IL-6, and TNF-α in the colonic tissues of mice (*p* < 0.05). Concurrently, the immunosuppressant cyclosporin A, allicin treatment, vitamin C treatment, and the combined treatment of vitamin C/allicin were all capable of significantly suppressing the elevation in the relative gene expression levels of IL-1β, IL-6, and TNF-α induced by DSS (*p* < 0.05). Moreover, the inhibitory efficacies of the separate or combined administration of vitamin C and allicin on the increment in the relative gene expression level of TNF-α induced by DSS surpassed that of cyclosporin A treatment.

#### 3.3.3. Impact of Vitamin C/Allicin Combined Treatment on the Fecal Microbial Diversity of DSS-Treated Mice

To scrutinize the effects of vitamin C treatment, high-dose allicin treatment, and the combined treatment of vitamin C/high-dose allicin on the microbial diversity of colonic chyme in DSS-induced mice, we resorted to 16S rRNA gene sequencing to analyze the fecal flora, thereby further elucidating the protective effect of the combined treatment of vitamin C/allicin on DSS-induced colitis.

**Figure 5:**
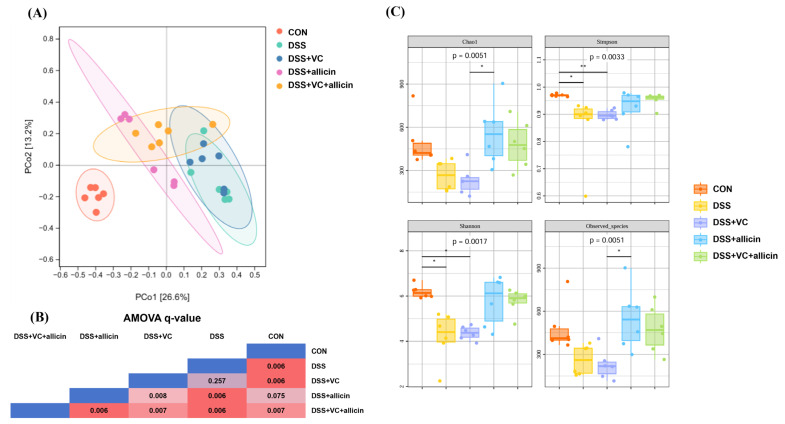
Effects of vitamin C and allicin on β-diversity (**A**,**B**) and α-diversity (**C**) of fecal microbiota in DSS mice. “*” represents a significant difference between groups, where * indicates *p* < 0.05, ** indicates *p* < 0.01.

In the context of microbial Beta (β) diversity, R and QIIME2 analysis software were utilized to conduct principal coordinate analysis (PCoA) and analysis of molecular variance (AMOVA) on the sequencing results of mouse fecal microorganisms. As presented in Figure 5A,B, in contrast to the control group, DSS treatment was capable of significantly modifying the structure of the mouse fecal microbial community (*p* < 0.01). Subsequent to the intervention with allicin or the combined intervention of allicin + vitamin C, successive and significant alterations also transpired in the structure of the fecal microbial community of DSS-treated mice (*p* < 0.01). However, the solitary intervention with vitamin C failed to induce any alteration in the structure of the fecal microbial community of DSS-treated mice (*p* > 0.05).

In the realm of microbial Alpha (α) diversity, the evenness and richness of the fecal microbial composition of DSS-treated mice were appraised by computing and analyzing the Shannon index, Simpson index, Chao1 index, and other relevant indices. As demonstrated in Figure 5C, when juxtaposed with the control group, DSS treatment engendered a significant diminution in the Simpson index and Shannon index of the mouse fecal microorganisms (*p* < 0.01). By contrast, no significant variations were discernible in the Simpson index and Shannon index of the fecal microorganisms in the allicin intervention group and the allicin + vitamin C combined intervention group (*p* > 0.05).

#### 3.3.4. Impact of Vitamin C/Allicin Combined Treatment on the Fecal Microbial Composition of DSS-Treated Mice

**Figure 6:**
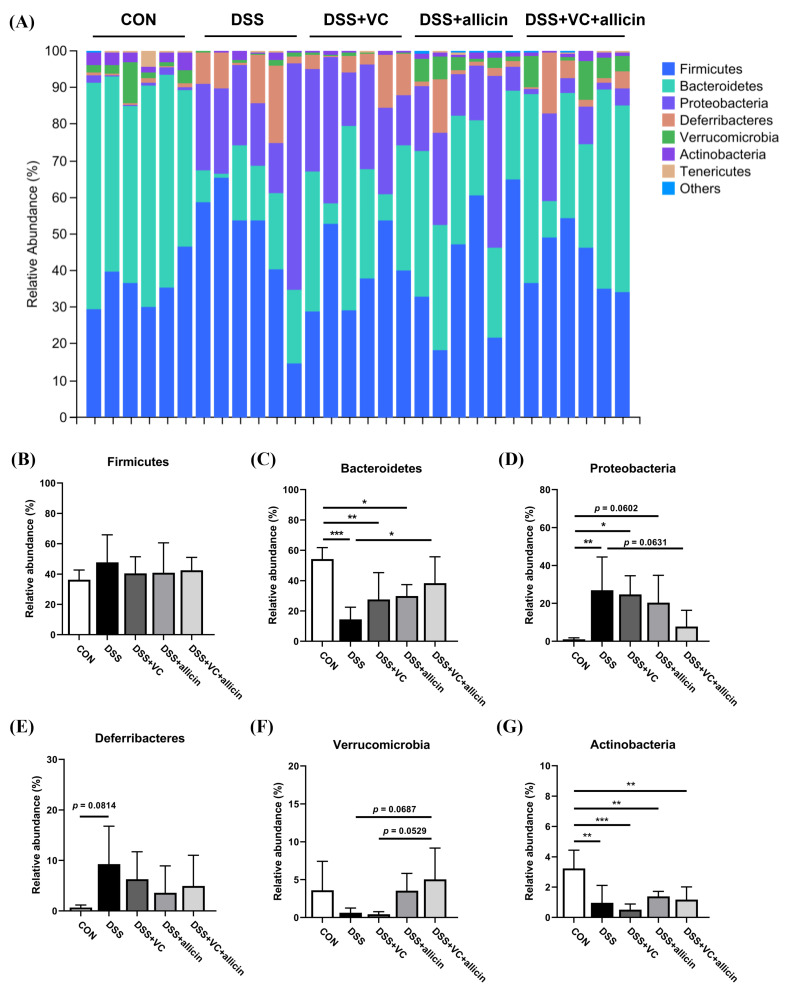
Effects of vitamin C and allicin on the intestinal flora of DSS mice. The intestinal flora structure at the phylum level (**A**), and the relative abundance of *Firmicutes* (**B**), *Bacteroidetes* (**C**), *Proteobacteria* (**D**), *Deferribacteres* (**E**), *Verrucomicrobia* (**F**), and *Actinobacteria* (**G**) in different treatment groups. Note: * indicates *p* < 0.05, ** indicates *p* < 0.01, and *** indicates *p* < 0.001.

After analyzing the composition of fecal microorganisms at the phylum level, it was found that, as shown in Figure 6A, the fecal microbial community mainly consisted of *Firmicutes*, *Bacteroidetes*, *Proteobacteria*, *Deferribacteres*, *Verrucomicrobia*, *Actinobacteria*, and *Tenericutes*. Through the statistical analysis of fecal microorganisms at the phylum level (Figure 6B–D), it was observed that DSS treatment had no impact on the relative abundance of *Firmicutes* but could significantly reduce the relative abundance of *Bacteroidetes* while significantly increasing the relative abundance of *Proteobacteria*. Neither allicin nor vitamin C intervention could influence the changes in *Bacteroidetes* and *Proteobacteria* in the feces of DSS-treated mice. However, the combined intervention of allicin and vitamin C could significantly alter the relative abundances of *Bacteroidetes* and *Proteobacteria* in the feces of DSS-treated mice, making them close to those of the control group. In addition, compared to the control group (Figure 6G), DSS treatment could also significantly decrease the relative abundance of *Actinobacteria* in the feces of mice (*p* < 0.01).

**Figure 7:**
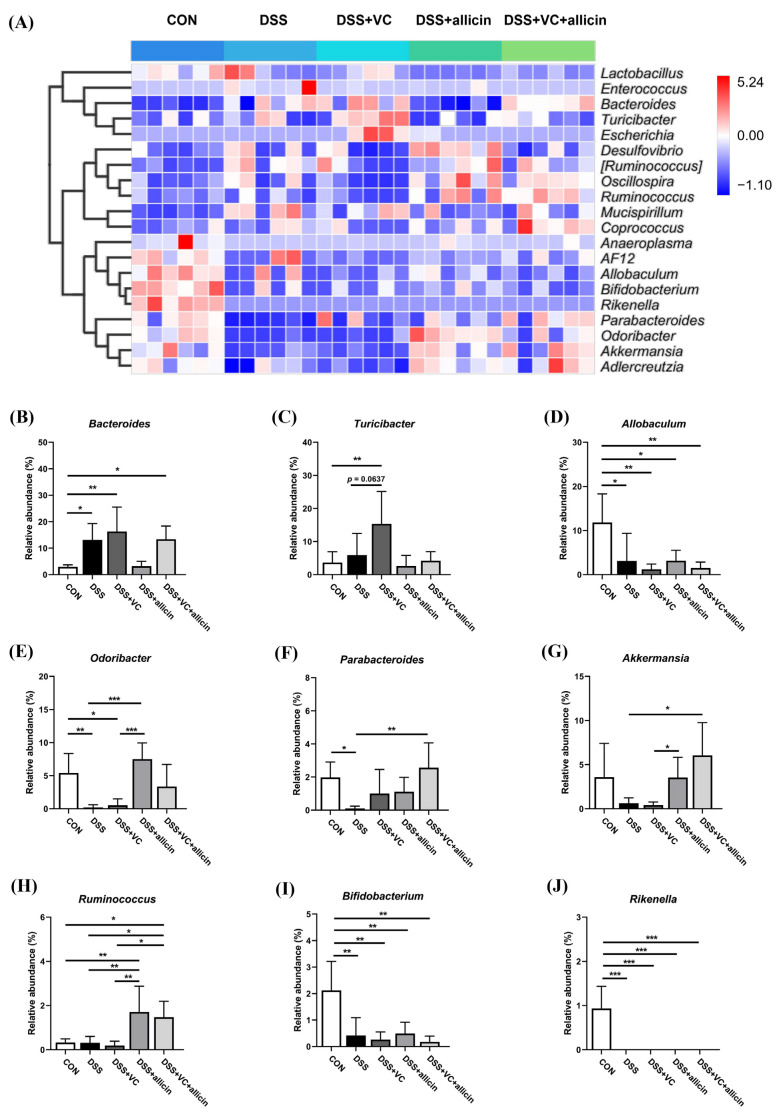
Effects of vitamin C and allicin on the intestinal flora of DSS mice. A heatmap at the genus level (**A**), and the relative abundance of *Bacteroides* (**B**), *Turicibacter* (**C**), *Allobaculum* (**D**), *Odoribacter* (**E**), *Parabacteroides* (**F**), *Akkermansia* (**G**), *Ruminococcus* (**H**), *Bifidobacterium* (**I**), and *Rikenella* (**J**) in the different treatment groups. Note: * indicates *p* < 0.05, ** indicates *p* < 0.01, and *** indicates *p* < 0.001.

To further reveal the impact of allicin and vitamin C on the fecal microbial composition of DSS-treated mice, heatmap clustering was employed to visually present the relative abundances of the main bacterial genera in the feces (the top 20 in terms of relative abundance). As shown in Figure 7A, DSS treatment led to an increase in the relative abundances of *Lactobacillus*, *Enterococcus*, *Bacteroides*, *Turicibacter*, *Escherichia*, *Desulfovibrio*, *Ruminococcus*, *Oscillospira*, *Ruminococcus*, *Mucispirillum*, and *Coprococcus*, while the relative abundances of *Anaeroplasma*, AF12, *Allobaculum*, *Bifidobacterium*, *Rikenella*, *Parabacteroides*, *Odoribacter*, *Akkermansia*, and *Adlercreutzia* decreased. Further statistical analysis revealed that the relative abundances of nine main bacterial genera underwent significant changes. As shown in Figure 7B–J, DSS treatment significantly decreased the relative abundances of *Allobaculum*, *Odoribacter*, *Parabacteroides*, *Bifidobacterium*, and *Rikenella* (*p* < 0.05). Vitamin C intervention tended to increase the relative abundance of *Turicibacter* in the feces of DSS-treated mice but had no significant regulatory effect on other main bacterial genera. Allicin intervention could significantly increase the relative abundances of *Odoribacter*, *Akkermansia*, and *Ruminococcus* in the feces of DSS-treated mice (*p* < 0.05). Under the combined intervention of allicin + vitamin C, the relative abundance of *Parabacteroides* in the feces of DSS-treated mice could also be increased, but it did not improve the reduction in the abundances of *Allobaculum*, *Bifidobacterium*, and *Rikenella* (*p* > 0.05).

#### 3.3.5. Impact of Vitamin C/Allicin Combined Treatment on the Fecal Microbial Functions of DSS-Treated Mice

**Figure 8:**
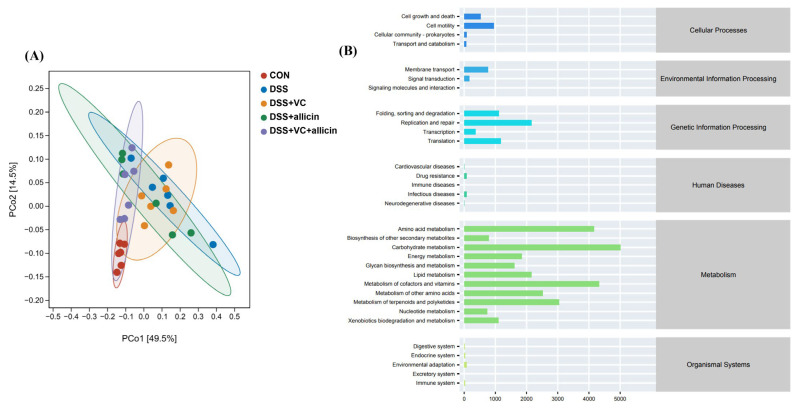
Principal component analysis (**A**) and KEGG enrichment analysis (**B**) of fecal microbial function of mice in different treatment groups.

To investigate whether allicin and vitamin C have an impact on the fecal microbial functions of DSS-treated mice, PICRUSt2 analysis was utilized to predict the microbial functions in the feces of the mice in each group. To specifically analyze the enrichment of fecal microbial functions in each group, the Kyoto Encyclopedia of Genes and Genomes (KEGG) was employed for functional enrichment analysis (as shown in Figure 8). Based on the principal component analysis of the abundance table of the unstratified sample metabolic pathways using normalized data, significant differences were found in the fecal microbial functions among different treatment groups. There were obvious differences in the functions of fecal microorganisms between the control group and the DSS treatment group, and the fecal microbial function composition of the DSS-treated mice with the combined intervention of allicin + vitamin C was clearly distinguishable from that of the DSS-treated mice, preliminarily indicating that the combined intervention of allicin + vitamin C effectively changed the intestinal microbial functions of DSS-treated mice. Through further enrichment analysis, it was found that the fecal microbial functions in each group were mainly enriched in directions such as metabolism, genetic information processing, cellular processes, and environmental information processing. Among them, the enrichment abundance in the metabolism direction was the highest, mainly enriched in pathways such as carbohydrate metabolism, the metabolism of cofactors and vitamins, amino acid metabolism, the metabolism of terpenoids and polyketides, the metabolism of other amino acids, and lipid metabolism.

**Figure 9:**
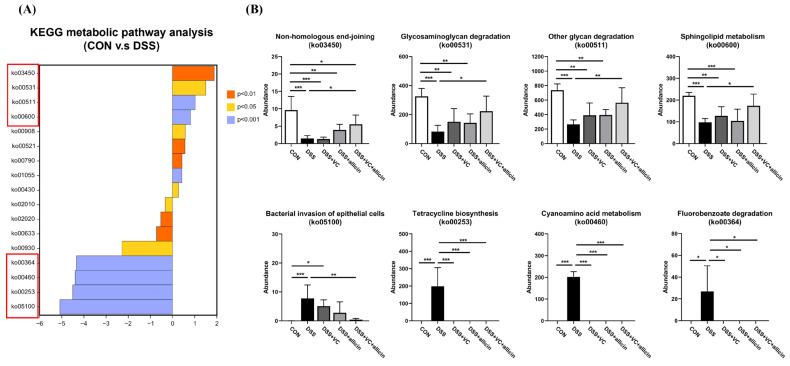
KEGG differential enrichment analysis of fecal microorganisms in control group and DSS group (**A**) and KEGG differential enrichment analysis of fecal microorganisms in different treatment groups (**B**). Note: * indicates *p* < 0.05, ** indicates *p* < 0.01, and *** indicates *p* < 0.001. ko03450: Non-homologous end-joining; ko00531: Glycosaminoglycan degradation; ko00511: Other glycan degradation; ko00600: Sphingolipid metabolism; ko00908: Zeatin biosynthesis; ko00521: Streptomycin biosynthesis; ko00790: Folate biosynthesis; ko01055: Biosynthesis of vancomycin group antibiotics; ko00430: Taurine and hypotaurine metabolism; ko02010: ABC transporters; ko02020: Two-component system; ko00633: Nitrotoluene degradation; ko00930: Caprolactam degradation; ko00364: Fluorobenzoate degradation; ko00460: Cyanoamino acid metabolism; ko00253: Tetracycline biosynthesis; ko05100: Bacterial invasion of epithelial cells.

To continue exploring the differential changes in the functions among the fecal microbial samples of each group, it was planned to first use the KEGG database to screen out the differential signaling pathways between the control group and the DSS treatment group, and then use statistical analysis to analyze the enrichment changes in the remaining groups in these differential signaling pathways to analyze the impact of different treatments on the changes in the fecal microbial functions of DSS-treated mice. As shown in Figure 9A, a total of 17 signaling pathways were differentially enriched. Among them, nine signaling pathways, including ko03450 non-homologous end-joining, ko00531 glycosaminoglycan degradation, ko00511 other glycan degradation, ko00600 sphingolipid metabolism, and ko00908 zeatin biosynthesis, were significantly elevated in the control group. Meanwhile, DSS treatment could significantly increase eight signaling pathways such as ko05100 bacterial invasion of epithelial cells, ko00253 tetracycline biosynthesis, ko00460 cyanoamino acid metabolism, and ko00364 fluorobenzoate degradation. The four signaling pathways with the largest fold changes in each group were selected for subsequent analysis. As shown in Figure 9B, DSS treatment significantly decreased the signaling pathways of non-homologous end-joining, glycosaminoglycan degradation, other glycan degradation, and sphingolipid metabolism (*p* < 0.05). Vitamin C + allicin could significantly improve the inhibitory effect induced by DSS on the above pathways (*p* < 0.05).

## 4. Discussion

Through a comparison of the impacts of drug treatment (cyclosporin A), allicin, and vitamin C, as well as the combined treatment of vitamin C/allicin on colitis, it was ascertained that the combined administration of vitamin C/allicin could mitigate colitis in mice, modulate and augment the composition of the intestinal flora in colitic mice, enhance the diversity of dominant flora, and foster the growth of beneficial bacteria.

### 4.1. Vitamin C/Allicin Can Ameliorate the Symptoms of Colitis in Mice

Colon length and the DAI constitute crucial parameters for assessing the severity of colitis [47]. Thus, based on the symptomatic phenotypic data pertaining to the treatment efficacies of the four treatment modalities for DSS model mice, it is conspicuously evident that the combined treatment of vitamin C/allicin could alleviate DSS-induced colitis in mice, intimating that the combined regimen of vitamin C/allicin might possess a comparable effect in relieving colitis symptoms to that of the immunosuppressant cyclosporin A. Nevertheless, the therapeutic outcomes of allicin or vitamin C when administered singularly were not statistically significant. Vitamin C has been documented to preclude, to a certain extent, the elevation of DAI scores induced by DSS [48]. Han et al. discovered that the oral administration of aged garlic could markedly attenuate DSS-induced colitis and diminish DSS-induced colon damage [26]. This finding deviates from the results of the current experiment, which could potentially be ascribed to the abbreviated treatment cycle of vitamin C and the disparate treatment modalities of allicin in the present study, in contrast to previous investigations. In the reported experiments wherein vitamin C was employed to alleviate colitis, the treatment duration spanned 5 weeks, whereas in this trial, the treatment period for vitamin C was merely 12 days; in the studies where allicin was utilized to mitigate colitis, the treatment protocol entailed pretreating with allicin for 8 days followed by DSS treatment for 6 days, while in this experiment, the selected approach was to administer allicin and DSS concurrently for 9 days, trailed by a 3-day solitary treatment with allicin. Consequently, the differential treatment cycles and modalities might underlie the discrepancy between the results of this experiment and those previously reported.

### 4.2. Vitamin C/Allicin Can Diminish the Expression of Inflammatory Factors in the Colon of Mice with Colitis

Lai et al. ascertained that DSS treatment could substantially augment the protein content of IL-6 and TNF-α [49]. In the assays designed to quantify IL-1β, IL-6, TNF-α, and the internal reference gene GAPDH in colon tissues, the outcomes indicating that all four treatment regimens reduced the expression levels of inflammatory cytokines suggested that allicin treatment, vitamin C treatment, and the combined treatment of vitamin C/allicin exerted a certain curative effect on mice with colitis, and their efficacies superseded those of the conventional immunosuppressant cyclosporin A for treating colitis. This is generally in concordance with the experimental findings of prior studies. For instance, allicin has been reported to suppress the increment in the protein level of IL-6 in the serum and colon of DSS-induced mice and to inhibit the elevation of the protein levels of IL-1β, IL-6, and TNF-α induced by LPS [26,50]. Vitamin C has also been shown to prevent DSS-induced colitis by regulating the production of IL-22 and IL-6 in mice [51]. In summary, it can be found that treatment with allicin and vitamin C alone or combined treatment with vitamin C/allicin can reduce the expression of inflammatory cytokines in the colon of colitis mice, and the therapeutic effect is better than that of cyclosporin A, a traditional immunosuppressive agent used to treat colitis.

### 4.3. Vitamin C/Allicin Can Effectively Regulate the Fecal Microbial Diversity, Composition, and Function in DSS-Treated Mice

The gut microbiota is regarded as one of the crucial factors in modulating host health [52]. Understanding the complexity of the relationship between the diversity, relative abundance, and function of the gut microbes and health will contribute to laying the foundation for new therapies currently under development. The results of our 16S rDNA sequencing experiments and bioinformatics analysis on the feces of DSS-treated mice indicate that the combined intervention of vitamin C/allicin can effectively improve the fecal microbial community structure in DSS-treated mice, promote the functions of beneficial bacteria, and subsequently alleviate the symptoms of colitis.

In terms of gut microbial diversity, both allicin intervention and the combined intervention of vitamin C/allicin can restore the evenness and richness of the gut microbial composition reduced by DSS induction. Notably, the vitamin C intervention did not alleviate the decrease in the gut microbial α-diversity of DSS-treated mice. It has been demonstrated in germ-free animal and antibiotic intervention models that an increase in gut microbiota diversity helps to maintain gut health [53]. Therefore, allicin intervention or the combined intervention of vitamin C/allicin would improve the gut damage in DSS-treated mice by upregulating the diversity of the gut microbiota.

In terms of the composition of the intestinal microbiota, the combination of allicin and vitamin C significantly increases the relative abundance of *Bacteroidetes* in the feces of DSS-induced colitis mice, bringing it closer to that of the control group. It has been demonstrated that certain genera within *Bacteroidetes*, such as *Allobaculum*, have the ability to alleviate intestinal inflammation through the fermentation of complex carbohydrates and the production of beneficial metabolites, specifically short-chain fatty acids (SCFAs) [54,55,56]. Similarly, at the genus level, we observed a notable increase in the relative abundance of *Akkermansia*, *Odoribacter*, *Ruminococcus*, and *Parabacteroides* following intervention with allicin or the combination of vitamin C and allicin. *Akkermansia*, a mucin-degrading bacterium belonging to *Verrucomicrobia*, has been extensively reported to enhance the epithelial cell layer integrity and contribute positively to reducing intestinal inflammation [57,58]. Additionally, *Odoribacter*, *Ruminococcus*, and *Parabacteroides* have all been identified as beneficial intestinal microorganisms, exhibiting negative correlations with the occurrence of intestinal inflammation [59,60,61]. Conversely, the relative abundance of potentially pathogenic *Proteobacteria* has significantly decreased following the combined intervention with allicin and vitamin C. These changes in the signature bacterial genera suggest that the combined intervention of vitamin C and allicin can regulate the alterations in the gut flora induced by DSS, primarily by promoting the colonization of beneficial bacteria, such as SCFA-producing strains, and reducing the proliferation of harmful bacteria, thereby alleviating intestinal inflammation to some extent.

In terms of gut microbial function, previous studies have shown that both the glycosaminoglycan degradation pathway and other glycan degradation pathways are related to the microbial fermentation and degradation of complex carbohydrates [62,63]. As the main metabolites of the microbial fermentation of complex carbohydrates, SCFAs play an important role in providing energy for intestinal epithelial cells, maintaining gut barrier function, and regulating gut immune function. Therefore, as mentioned above, DSS treatment would limit the ability of gut microbes to ferment and degrade complex carbohydrates, reduce the production of SCFAs, and thus exacerbate gut inflammation. Under the combined intervention of vitamin C/allicin, the reduction degree of the above pathways induced by DSS can be alleviated, indicating that allicin + vitamin C can relieve gut inflammation by effectively improving the ability of gut microbes in DSS-treated mice to ferment and degrade complex carbohydrates. In addition, DSS treatment significantly increased the signaling pathways of the bacterial invasion of epithelial cells, tetracycline biosynthesis, cyanoamino acid metabolism, and fluorobenzoate degradation. This indicates that DSS treatment would increase the risk of gut bacteria infecting intestinal epithelial cells. The combined intervention of vitamin C/allicin can significantly reduce the enrichment of the bacterial invasion of epithelial cell signals and reduce the risk of gut infection.

This study preliminarily explored the repair effects of vitamin C and allicin on DSS-induced colitis in mice from aspects such as phenotype, inflammatory cytokines, and microbial communities.

## 5. Conclusions

In this study, the effects of fertilizer and varying concentrations of anaerobic fermentation liquid (AFL) on the growth and vitamin C content of Bok choy were investigated. The results demonstrated that, compared to the control fertilizer group (CF group), AFL significantly enhanced the growth and development of Bok choy, including increasing the plant height, leaf number, fresh weight, and fresh weight of the edible portion. As the concentration of AFL increased, the growth characteristics of Bok choy exhibited a concentration-dependent trend, with the highest yield observed in the AFL-2 group (8.43 kg/m^2^). Additionally, the AFL-1 group exhibited the highest vitamin C content (70.83 mg/100 g). Subsequently, response surface methodology was employed to optimize the microwave-assisted organic solvent extraction process for vitamin C. The findings indicated that the optimal conditions for the microwave-assisted extraction of vitamin C from a 2% citric acid solution were as follows: a microwave power of 313 W, a microwave time of 1.3 min, and a liquid–solid ratio of 16.4:1 *v*/*w*, resulting in an extraction rate of 90.77%. Finally, this study elucidated the protective effect of combined vitamin C/allicin therapy on DSS-induced colitis in mice. The results showed that the combination of vitamin C and allicin significantly alleviated inflammatory responses and reduced the risk of intestinal infection in colitis mice by modulating the structure of the gut microbiota, inhibiting the expression of inflammatory factors, and reducing bacterial invasion signals in epithelial cells. Moreover, this study concluded that the therapeutic efficacy of the vitamin C/allicin combination on DSS-induced colitis was superior to that of either allicin or vitamin C alone. This research provides a valuable theoretical foundation and technical support for the utilization of agricultural waste resources, the efficient extraction of natural products, and the treatment of inflammatory bowel disease. Further research is needed to elucidate the specific functions and anti-inflammatory mechanisms of each type of responsive bacteria. Future studies could also delve deeper into identifying the epigenetic metabolites and metabolic pathways that play a protective role in ulcerative colitis through targeted metabolomics and SCFA detection.

## Figures and Tables

**Table 1:** Chemical composition of AFL after treatment.

Parameters	Value
Total Solids (TS in g/L)	2.02 ± 0.12
Total Organic Compounds (TOC in g/L)	1.65 ± 0.20
Humic Acids (HA in g/L)	1.32 ± 0.08
Chemical Oxygen Demand (COD in mg/L)	1307.58 ± 6.99

Note: The total solids content and chemical oxygen demand are derived from our previous studies [21].

**Table 2:** Experimental group setting of Bok choy (L/plot (3.52 m^2^)).

Name	Seedling Acceleration Stage	Strong Seedling Period	Harvesting Stage
BC	2.33	0	0
CF	0.0275	0.0355	0.0355
AFL-0.5	5.745912	7.39817	7.39817
AFL-1	11.49182	14.79634	14.79634
AFL-2	22.98365	29.59268	29.59268

Note: BC was the blank control group, where only organic fertilizer (mainly biogas residue) was applied as the base fertilizer, and no additional fertilizer was applied during the growth period. CF was the fertilizer group, where only urea was applied (about 46.6% N). AFL-0.5, AFL-1, and AFL-2 were treated with anaerobic fermentation liquid, and their total nitrogen contents were 0.5, 1, and 2 times that of the CF group, respectively.

**Table 3:** Factors and level setting in the design of the response surface for vitamin C extraction.

Factors	Code Character	Level		
		−1	0	1
Microwave power (W)	A	280	380	480
Microwave time (min)	B	1	1.5	2
Liquid-to-material ratio (*v*/*w*)	C	10	15	20

**Table 4:** Primer sequences used for the quantification of mRNA expression by real-time PCR.

Gene	Primer Pairs Sequence	Product Length (bp)
*IL-1β*	F: TGCCACCTTTTGACAGTGATG	138
	R: TGATGTGCTGCTGCGAGATT	
*IL-6*	F: TAGTCCTTCCTACCCCAATTTCC	76
	R: TTGGTCCTTAGCCACTCCTTC	
*TNF-α*	F: CCCTCACACTCAGATCATCTTCT	61
	R: GCTACGACGTGGGCTACAG	
*GAPDH*	F: AGGTCGGTGTGAACGGATTTG	123
	R: TGTAGACCATGTAGTTGAGGTCA	

Notes: IL-1β: interleukin-1β; IL-6: interleukin 6; TNF-α: tumor necrosis factor α; GAPDH: internal reference gene.

**Table 5:** Experimental verification results of optimal microwave treatment conditions.

	Microwave Power (W)	Microwave Time (min)	Liquid–Solid Ratio (*v/w*)	Extraction Rate (%)
1	413	1.3	16.4:1	90.51
2	413	1.3	16.4:1	91.25
3	413	1.3	16.4:1	90.29
Average value				90.68

## Data Availability

The original contributions presented in this study are included in the article/Supplementary Materials. Further inquiries can be directed to the corresponding authors.

## Supplementary Materials

The following supporting information can be downloaded at: https://www.mdpi.com/article/10.3390/foods14050785/s1, Table S1 Properties of AFL after treatment; Table S2 Experimental design and results of Box-Behnken; Table S3 Results of Quadratic vs. 2FI model on the response surface of vitamin C extraction rate.
