# Supplementary material for: Impact of Anaerobic Fermentation Liquid on Bok Choy and Mechanism of Combined Vitamin C from Bok Choy and Allicin in Treatment of DSS Colitis
Source: Foods. 2025 Feb 25;14(5):785. doi: 10.3390/foods14050785 (PMC11899586; doi:10.3390/foods14050785)
Supplement: Supplementary file 1 [file foods-14-00785-s001.zip › foods-3468967-supplementary.pdf]

Supplementary Materials

Impact of anaerobic fermentation liquid on Bok choy and mechanism of combined vitamin C from Bok choy and allicin in treatment of DSS colitis

Junhui Pan [1,†], Kaitao Peng [1,†], Roger Ruan [2], Yuhuan Liu [3,*] and Xian Cui [1,*]

[1] State Key Laboratory of Food Science and Resources, Engineering Research Center for Biomass Conversion, Ministry of Education, Nanchang University, Nanchang 330047, China; panjunhui@ncu.edu.cn (J.P.); pkt1811826@163.com (K.P.)

[2] Center for Biorefining and Department of Bioproducts and Biosystems Engineering, University of Minnesota, St. Paul, MN 55108, USA; ruanx001@umn.edu

[3] Chongqing Research Institute of Nanchang University, Chongqing 402660, China

* Correspondence: liuyuhuan@ncu.edu.cn (Y.L.); cuixian@ncu.edu.cn (X.C.)

† These authors contributed equally to this work.

Table S1 Properties of AFL after treatment.

Parameters	Value
Total nitrogen (mg/L)	1070 ± 15
Total salt (g/L)	2.45 ± 0.09
Electric conductivity (ms/cm)	15.77 ± 1.25
Odor concentration	577.76 ± 5.62
Total phosphorus (mg/L)	15.97 ± 1.06
K (mg/L)	2078.50 ± 19.56
Mg (mg/L)	14.61 ± 1.36
Ca (mg/L)	140.32 ± 4.92
Cu (mg/L)	3.12 ± 0.09
Zn (mg/L)	11.43 ± 0.92
As (mg/L)	18.05 ± 0.85
Se (mg/L)	0.24 ± 0.02

Note: The data in the table is consistent with those reported in our previous study [1].

Table S2 Experimental design and results of Box-Behnken.

	Microwave power（W）	Microwave time（min）	Liquid-solid ratio（*v/w*）	Extraction rate（%）
1	380	1.5	15	89.18
2	380	2	20	78.91
3	380	1	10	77.36
4	480	1	15	66.73
5	380	1.5	15	88.09
6	480	1.5	10	72.18
7	280	1.5	20	85.18
8	380	1.5	15	87.27
9	380	1.5	15	89.45
10	380	1.5	15	88.36
11	280	1	15	89.45
12	480	1.5	20	77.27
13	480	2	15	76.55
14	380	1	20	81.55
15	280	2	15	71.55
16	280	1.5	10	79.55
17	380	2	10	71.09

Table S3 Results of Quadratic vs 2FI model on the response surface of vitamin C extraction rate.

Source of variance	Sum of squares	Degree of freedom	Mean square error	F	p-value	significance
model	886.40	9	98.49	168.18	<0.0001	**
A- Microwave power	136.12	1	136.12	232.45	<0.0001	**
B- Microwave time	36.08	1	36.08	61.61	0.0001	**
C- Liquid-solid ratio	64.58	1	64.58	110.28	<0.0001	**
AB	192.10	1	192.10	328.03	<0.0001	**
AC	0.0729	1	0.00729	0.1245	0.7346	—
BC	3.29	1	3.29	5.63	0.0495	*
A2	129.29	1	129.29	220.77	<0.0001	**
B2	198.07	1	198.07	338.23	<0.0001	**
C2	80.91	1	80.91	138.17	<0.0001	**
Residual error	4.10	7	0.5856	—	—	—
Missing fit	1.04	3	0.3461	0.4523	0.7297	—
Absolute error	3.06	4	0.7653	—	—	—
Total deviation	890.50	16	—	—	—	—
R^2：0.9954 Adjusted R^2：0.9895						
Predicted R^2：0.9760 Adeq Precision：37.6709						

Note: ** means extremely significant ($p < 0.01$), * means significant ($p < 0.05$)

References

1. Pan, J.; Shen, J.; Zhou, Z.; Xin, Y.; Huang, Z.; Xiong, J.; Liu, Y.; Cui, X.; Liu, Y. Sustainable Management of Biogas Slurry Discharge in Biogas Engineering: As a Chemical Fertilizer Substitute for Garlic Cultivation. *BioRes* **2024**, *20*, 790–808, doi:10.15376/biores.20.1.790-808.
